# From Pyelonephritis to Vasculitis: A Challenging Diagnosis of Renal-Limited Vasculitis Presenting As Acute-on-Chronic Renal Failure

**DOI:** 10.7759/cureus.87542

**Published:** 2025-07-08

**Authors:** Sopio Motsonelidze, Levan Gulua

**Affiliations:** 1 Internal Medicine, Aiken Regional Medical Center, Aiken, USA

**Keywords:** anca associated vasculitis, myeloperoxidase (mpo), pyelonephritis, rapidly progressive glomerulonephritis (rpgn), renal-limited vasculitis (rlv)

## Abstract

Renal-limited vasculitis (RLV) is a rare form of anti-neutrophil cytoplasmic antibody (ANCA)-associated vasculitis. The kidneys are the primary, and often the only, organs affected by this disease. A 66-year-old white male presented with hematuria and acute-on-chronic renal failure. Computed tomography (CT) of the abdomen and pelvis revealed left-sided pyelonephritis/cystitis. Urinalysis showed elevated white blood cell (WBC) and red blood cell (RBC) counts, with 1+ bacteria and 3+ proteinuria. Serum creatinine was 5.6 mmol/L, and estimated glomerular filtration rate (eGFR) was 10 mL/min/1.73 m². The patient was admitted with a diagnosis of pyelonephritis and treated with antibiotics. The patient showed no clinical or laboratory improvement with antibiotics. Urine culture was negative, and the urine protein-to-creatinine ratio indicated nephrotic-range proteinuria. Further tests, including an antinuclear antibody (ANA) panel, anti-cytoplasmic antineutrophil cytoplasmic antibodies (ANCA), and urine and serum electrophoresis were ordered. Anti-myeloperoxidase (MPO) antibody was positive. The patient subsequently underwent a kidney biopsy, which revealed pauci-immune crescentic glomerulonephritis, severe interstitial fibrosis, severe tubular atrophy, and arterionephrosclerosis. The patient was diagnosed with Myeloperoxidase (MPO)-associated RLV and was started on pulse dose steroids, prednisone 1 milligram per kilogram (mg/Kg) daily, and weekly Rituximab 375 milligrams per square meter (mg/m²). The patient received two doses of Rituximab while inpatient, with significant improvement in kidney function. This case highlights the importance of considering the possibility of underlying vasculitis in cases of refractory renal failure and proteinuria, even when initial symptoms, laboratory tests, and imaging suggest an infection.

## Introduction

Renal-limited vasculitis (RLV) is a pauci-immune vasculitis that primarily affects the kidney and is characterized by necrotizing glomerulonephritis with minimal or no immune complex deposits. The vast majority of patients with RLV test positive for anti-neutrophil cytoplasmic antibody (ANCA), with 75 to 80 percent having myeloperoxidase-specific anti-neutrophil cytoplasmic antibody (MPO-ANCA) [1-3].

As the histopathologic findings in the kidney are identical to those of glomerulonephritis in GPA or MPA, and because some patients who initially present with disease limited to the kidney eventually show extrarenal manifestations of either GPA or MPA, ANCA-positive pauci-immune necrotizing glomerulonephritis is thought to be a part of the spectrum of granulomatosis with polyangiitis (GPA) and microscopic polyangiitis (MPA) [3,4].

Although renal-limited vasculitis (RLV) is a rare condition, it can be misdiagnosed as pyelonephritis due to overlapping symptoms and the often insidious presentation of kidney involvement. Here, we present a patient who was initially admitted for treatment of pyelonephritis and eventually was diagnosed with RLV.

## Case presentation

A 66-year-old white male with a past medical history significant for hypertension(on Lisinopril 10 mg), chronic kidney disease (CKD), hyperlipidemia (on Atorvastatin 10mg), and nephrolithiasis presented with hematuria and was admitted for acute kidney injury(AKI) on top of chronic kidney disease (CKD). Lisinopril was discontinued upon admission due to AKI associated with CKD. Initial workup showed a white blood cell count of 12.3 mm^3^, hemoglobin of 10.2 g/dL, creatinine of 5.6 mg/dL (baseline: 1.4 mg/dL), estimated glomerular filtration rate (eGFR) of 10 mL/min/1.73m^2^ (baseline: 43 mL/min/1.73m^2^), and potassium of 5.3 mmol/L (Table 1). Urinalysis results showed positive leukocyte esterase, with 3+ blood, a urine red blood cell count of 919, a urine white blood cell count of 143, and 1+ bacteria.

**Table 1:** Result of blood test

Test	Reference range	Result
White blood cells	3.3-10.7 x 10^3^/μL	12.3
Hemoglobin	13.6-17.3 x g/dL	10.2
Platelets	140-425 x 10^3^/μL	222
Sodium	137-145 mmol/L	133
Potassium	3.50-5.10 mmol/L	5.3
Chloride	96-107 mmol/L	103
CO_2_ (Carbon Dioxide)	22-30 mmol/L	16
Blood Urea Nitrogen	7-20 mg/dL	70
Creatinine	0.6-1.2 mg/dL	5.6
Estimated glomerular filtration rate	90 mL/min/1.73 m² or higher	10

**Figure 1:**
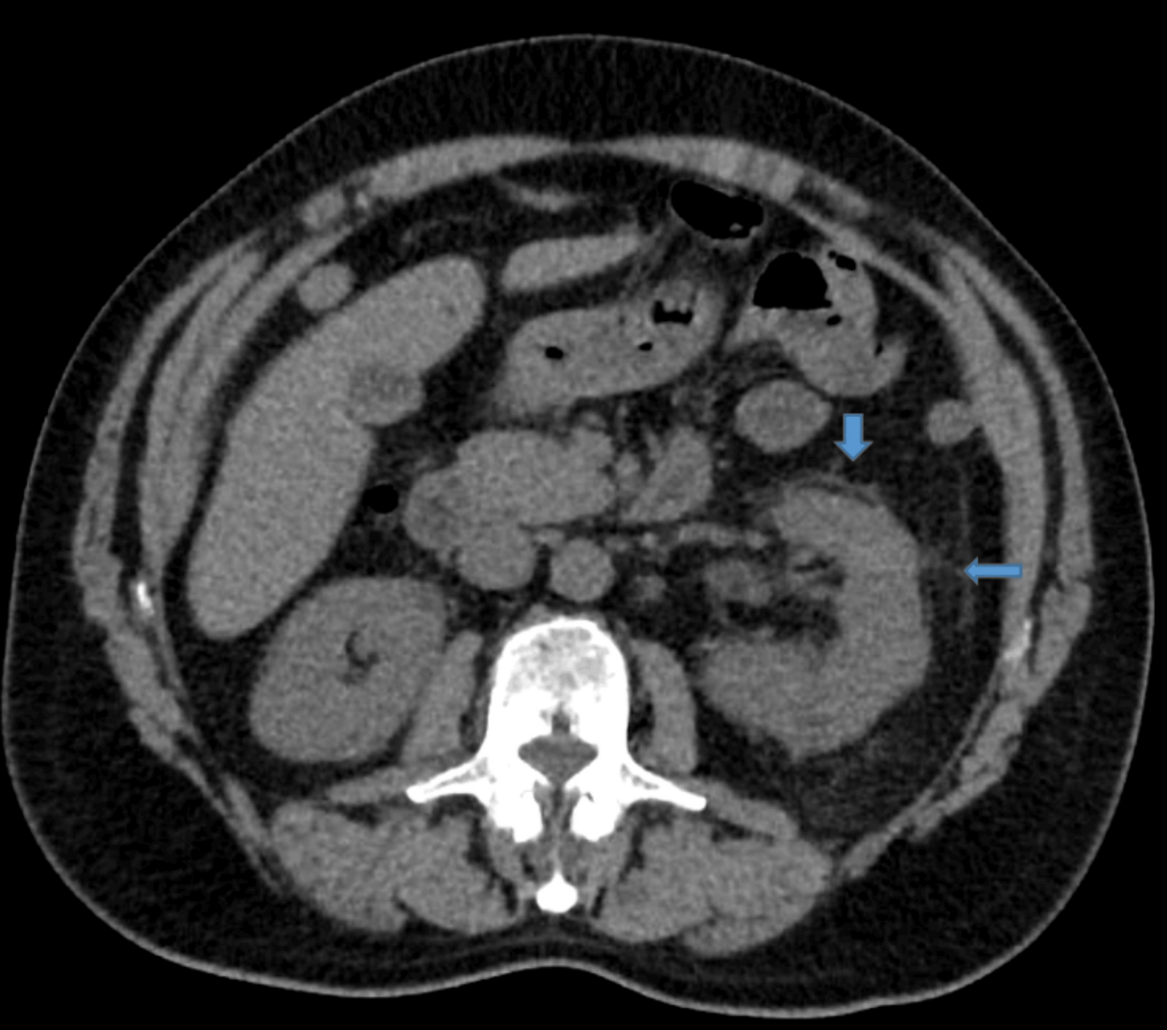
Computed tomography of the abdomen and pelvis without contrast showed left perinephric edema (blue arrow), consistent with left-sided pyelonephritis.

Computed tomography of the abdomen and pelvis was concerning for left-sided pyelonephritis (Figure 1). The patient was placed on piperacillin-tazobactam, and nephrology was consulted. The next day, the antibiotics were changed from piperacillin-tazobactam to levofloxacin. The patient showed no clinical or laboratory improvement with antibiotic treatment. Serum creatinine peaked at 8.9 mg/dL and remained elevated until appropriate interventions were initiated. Urine culture results were negative. As per nephrology recommendations, antibiotics were discontinued. The urine protein-to-creatinine ratio showed nephrotic-range proteinuria of 4600 mg/g. The 24-hour urine protein test confirmed nephrotic-range proteinuria at 5,712 mg/24 hours (Table 2). An antinuclear antibody (ANA) panel, complement level, urine and serum electrophoresis, anti-glomerular basement membrane (GBM) antibodies, hepatitis panel, proteinase three antibody, Perinuclear P-ANCA, cytoplasmic C-ANCA, and atypical pANCA were unremarkable. Antimyeloperoxidase(MPO) antibodies were positive (>8 ) (Table 3).

**Table 2:** Urine Chemistry

Test	Reference range	Result
Urine Total Protein/24 Hr	42-225 mg/24hr	5712
Urine Protein/Creatinine	0-200 mg/g	4600

**Table 3:** Anti-neutrophil cytoplasmic antibodies (ANCA)profile Abs=Antibodies

Test	Reference range	Result
Antiproteinase 3 (PR-3) Abs.	0.0-0.9 units	<0.2
Perinuclear (P-ANCA)	<1:20 Titer	<1:20
Cytoplasmic (C-ANCA)	<1:20 Titer	<1:20
Atypical pANCA	<1:20 Titer	<1:20
Antimyeloperoxidase (MPO) Abs.	0.0-0.9 units	>8.0

**Figure 2:**
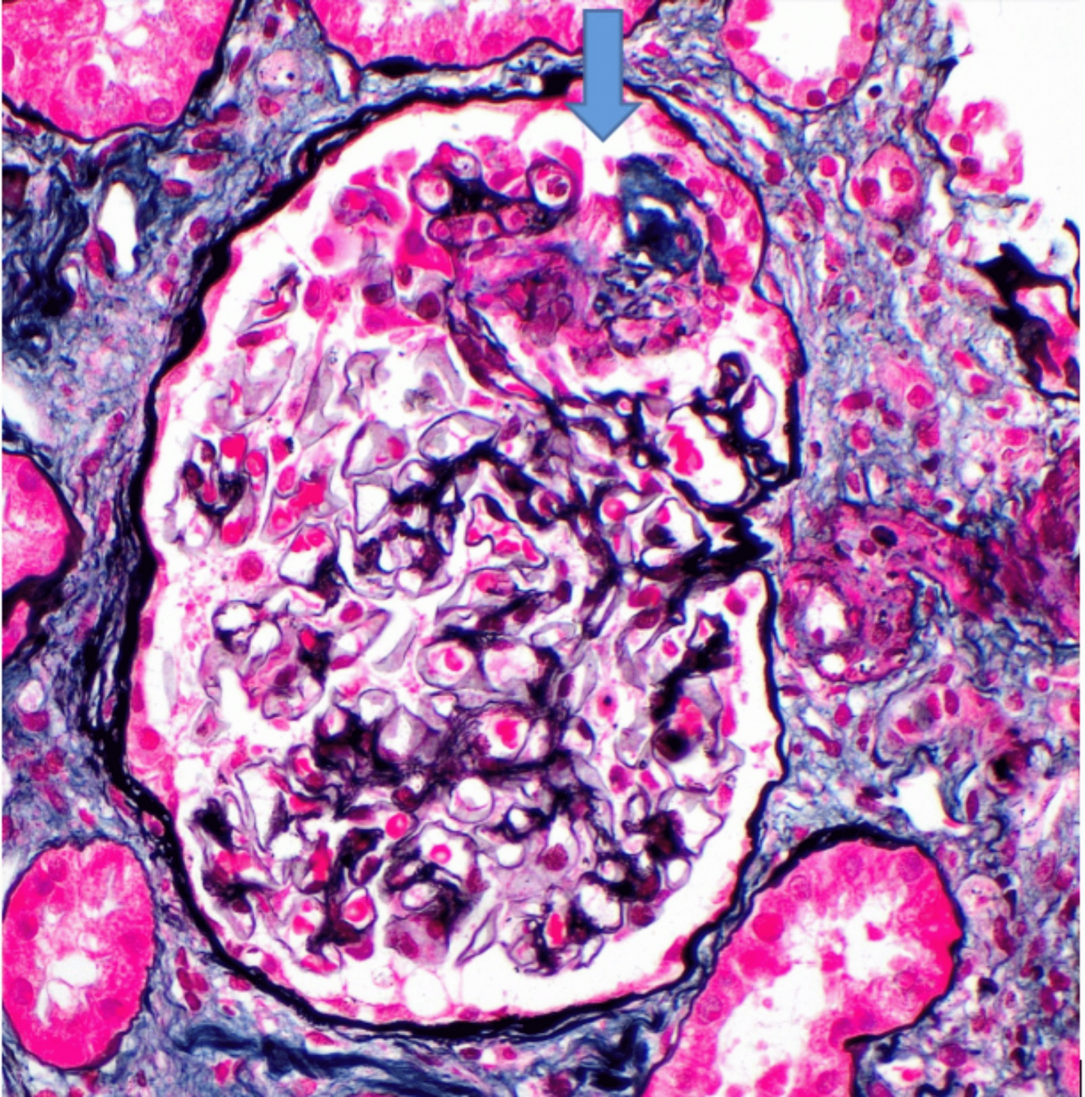
Glomerulus stained for Jones methenamine silver shows a segmental cellular/fibrocellular crescent (blue arrow).

**Figure 3:**
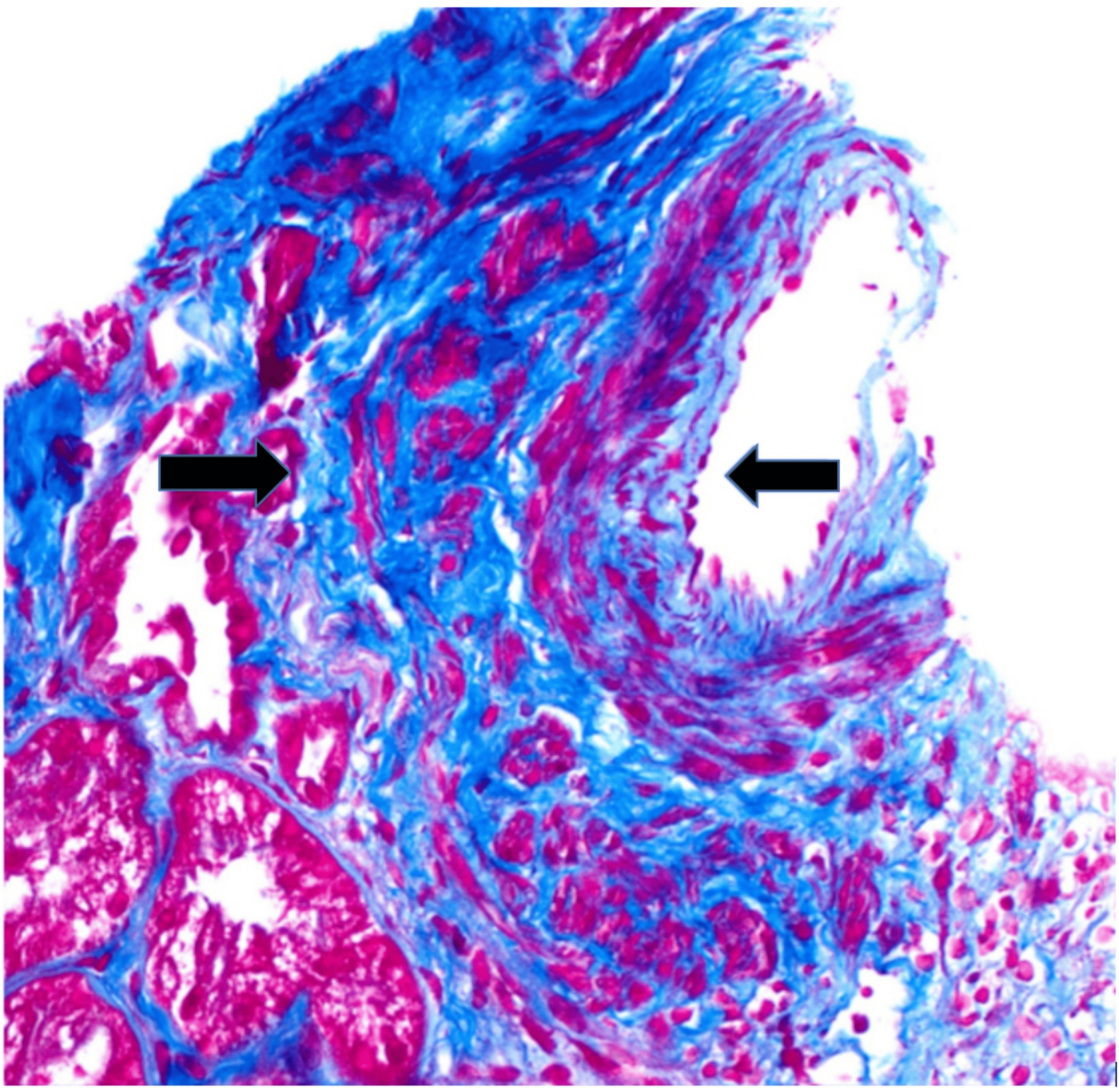
Masson's Trichrome Stain shows arterial intimal and medial fibrosis (black arrows).

The patient underwent a kidney biopsy due to concerns about glomerulonephritis. Renal biopsy showed pauci-immune, crescentic glomerulonephritis, severe interstitial fibrosis, and severe tubular atrophy with arterionephrosclerosis (Figures 2, 3). Based on clinical presentation, laboratory findings, and renal biopsy, the patient was diagnosed with MPO-associated RLV. The patient was started on prednisone 1 mg/kg daily and Rituximab 375 mg/m² weekly. He received two doses of rituximab while inpatient, with significant improvement in kidney function (creatinine improved from 8.9 mg/dL to 4.4 mg/dL). The patient was discharged home with a follow-up appointment scheduled with the department of nephrology. At the nephrology clinic, the patient underwent repeat ANCA testing, which showed positive anti-myeloperoxidase antibodies and a weakly positive p-ANCA titer of 1:80.

## Discussion

Rapidly progressive glomerulonephritis (RPGN) is a clinical condition characterized by a rapid decline in kidney function, typically occurring within weeks or a few months, and accompanied by characteristic glomerular disease features in the urinalysis [5,6].

When referring to crescentic glomerulonephritis (GN), the term RPGN typically refers to one of three general mechanisms of glomerular injury: pauci-immune necrotizing and crescentic GN, immune complex-mediated injury, or anti-glomerular basement membrane (GBM) disease [5,6].

Based on immunofluorescence or electron microscopy, a necrotizing GN with few or no immune deposits is classified as pauci-immune necrotizing and crescentic GN. Systemic vasculitis, which affects the ears, nose, throat, skin, and lungs, is often associated with pauci-immune necrotizing and crescentic glomerulonephritis. Renal-limited vasculitis is the term used to describe a condition that is restricted to the kidneys. Many patients with RLV will develop systemic symptoms of microscopic polyangiitis (MPA) or granulomatosis with polyangiitis (GPA), and the majority of these patients are ANCA-positive [6]. People with kidney-limited pauci-immune crescentic glomerulonephritis (GN) that is ANCA-negative are included in this spectrum. They might have comparable prognoses, kidney biopsy results, and clinical characteristics.

For patients who present with clinical findings suggestive of RPGN, a precise and prompt diagnosis is crucial. Unless it is contraindicated, patients should have a kidney biopsy and the necessary serologic tests ordered on a stat or urgent basis. Serologic tests include, but are not limited to, ANCA, anti-GBM antibodies, complement component assays, antinuclear antibodies, and cryoglobulins. Additional tests should be considered based on the clinical history, examination, and biopsy results [5,6].

For the majority of RPGN patients, the first-line treatment usually consists of pulse methylprednisolone, daily oral prednisone, oral or intravenous cyclophosphamide, or rituximab, and, in certain situations, plasmapheresis. Minimizing the extent of irreversible kidney damage requires early diagnosis based on kidney biopsy and serologic testing, as well as early initiation of the proper treatment [6,7].

Our patient received treatment based on the Rituximab for ANCA-Associated Vasculitis (RAVE) trial (induction therapy with rituximab 375 mg/m² per week for four weeks), resulting in significant improvement in kidney function [8].

## Conclusions

This case report highlights the successful diagnosis and management of a 66-year-old male with MPO-associated renal-limited vasculitis, a rare form of ANCA-associated vasculitis (AAV) affecting only the kidneys. The patient presented with acute Kidney Injury and chronic kidney disease, initially suggestive of a urinary tract infection. Still, the lack of response to antibiotics, combined with nephrotic-range proteinuria and positive MPO-ANCA, raised suspicion for glomerular disease. The diagnosis was confirmed by a kidney biopsy showing pauci-immune, crescentic glomerulonephritis, severe interstitial fibrosis, and severe tubular atrophy, characteristic findings of AAV. Prompt treatment with high-dose steroids and Rituximab led to significant improvement in kidney function, emphasizing the importance of early diagnosis and aggressive immunosuppressive therapy in RLV.
